# Sudden cardiac death in congenital heart disease—a narrative review and update

**DOI:** 10.3389/fcvm.2025.1539958

**Published:** 2025-04-30

**Authors:** Frank Han

**Affiliations:** ^1^ Division of Pediatric Cardiology, Department of Pediatrics, University of Illinois, Chicago, IL, United States; ^2^ Division of Pediatric Cardiology, Department of Pediatrics, Connecticut Children's Medical Center, Hartford, CT, United States

**Keywords:** sudden cardiac arrest, adult congenital heart disease, grown up congenital heart disease, pediatric cardiology, Tetralogy of Fallot

## Abstract

The spectrum of congenital heart disease is extremely varied, from simple septal defects all the way up to complex heterotaxy with multiple overlapping congenitally malformed regions of the heart. While surgical repair has come a long way since the first congenital cardiac surgery, a B-T-T shunt, an unmet need remains as the population continues to experience sudden cardiac arrest at a greater rate than the general population. Many advances in pacing and cardioversion have occurred to address bradyarrhythmias and tachyarrhythmias, but these carry their own adverse effect profile and limitations. This review aims to survey the field, summarize advances, and provide suggestions for future research directions.

## Introduction

The congenitally malformed heart can have many different electrophysiological issues in isolation or in combination, depending on the malformation. While sudden cardiac arrest continues to occur in the acquired heart disease population, it occurs at a greater rate in the congenital heart disease population. Comprehensive reviews of heart failure relevant to congenital heart disease and detailed electrophysiological studies of the congenitally malformed heart are described elsewhere; this review will address those topics in broad strokes. This narrative review aims to describe the current understanding of common arrhythmias and sudden cardiac arrest in congenital heart disease along with aspects unique to the congenitally malformed heart. The review will begin with an overview of key aspects of cardiac embryology, pointing out the cause of selected congenital heart lesions from their embryological origins. The embryological origins of the conduction system will be discussed. Next, common congenitally malformed hearts with their unique mechanisms of cardiac arrest will be introduced. Finally, treatments and new developments in arrhythmia management will be discussed.

## Embryologic development of the normal heart

As organisms evolved beyond the multicellular stage, a need grew for there to be a specific organ system dedicated to removing waste products and sending in nutrients/oxygen. The cardiovascular system begins as a tube, and folds gradually/adds on tissue in various regions based upon local molecular signals to become the fully developed heart (1). Different organisms have slightly different versions of a fully developed heart, e.g., frogs normally carry three chambers (2), but in humans the process of folding and development normally completes by eight weeks of gestation. The heart then typically grows bigger carrying the same configuration that it had reached, with limited capacity for regeneration (3). In limited laboratory studies, the capacity of the human heart to regenerate appears to be mostly limited to the first few weeks after birth (4). The embryonic heart, at least according to chick models, retains the ability to regenerate externally damaged tissue through the proliferation of adjacent undamaged cardiomyocytes (5). In another mouse model, up to 60% of embryonic cardiomyocytes could be destroyed by day 9 of development, with subsequent complete restoration of cardiac structure and function (6). Attempts to restore the ability of cardiomyocytes to regenerate in adult hearts generally center around attempts to replenish stem cells, reprogram stem cells, or reprogram existing cardiac fibroblasts (7). A variety of biomedical engineering techniques are also attempting to discover the most effective way to deliver new cells into injured regions of the heart (8). At current no licensed clinically available tool for regenerating cardiomyocytes is available.

## Development of the normal conduction system

**Figure 1:**
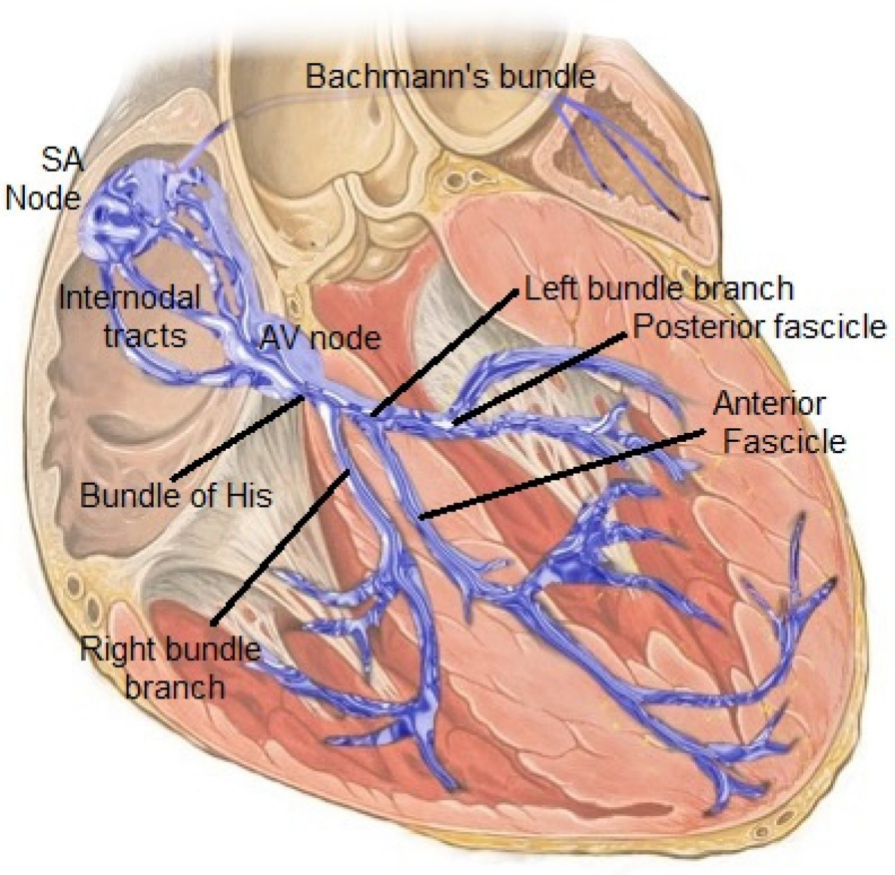
The normal cardiac conduction system. Reproduced with permission from “Cardiac conduction system” by Npatchett, licensed under CC BY-SA 4.0.

In the fully formed heart, the conduction system's major components are the sinoatrial node, atrioventricular node, and left/right bundle branches (9) (Figure 1). Conduction tissue cannot be directly seen with the naked eye, although a good idea of its position can be approximated during surgery using anatomical landmarks such as the right ventricular moderator band and triangle of Koch. Multiple complex molecular, cellular, and connective tissue intricacies define the structure and function of these primary components.

The sinoatrial node in both the normal and congenitally malformed heart is responsible for starting a cardiac electrical impulse; these cells are unique in their ability to self-depolarize due to a time-varying baseline current (10) that produces a heart rate that normally varies with activity level (and with the animal). The current is rapidly transmitted across both atria and reaches the left atrium via the Bachmann's bundle. The current is slowed by the AV node, and normally insulated from the ventricles by the electrically insulating atrioventricular valves/fibrous trigone. This temporary slowing, forming much of the P-R interval on ECG, is necessary to allow a coordinated contraction of the ventricles after the atrial contraction. It also protects the ventricle from accidental arrhythmias that may be triggered by errant atrial beats. The heartbeat is finally transmitted towards the ventricles across the His bundle and left/right bundles. In the congenitally malformed heart, the conduction system still follows these general rules, but can present unusual anatomical variants like dual SA nodes, dual -AV nodes with elongated bundles in L-transposition of the great arteries, or live close to the boundary of a ventricular septal defect, which makes its closure more tricky (11).

The earliest embryologic iteration of the heart is a cellular cylinder, and as such, the earliest electrical depolarizations travel from one end to the other in a sinusoidal pattern, generating a repeating wave of peristalsis (12). Even before the definitive SA node forms, cells corresponding to the “entrance” of the primitive cardiac tube have a higher rate of depolarization than the other cells, therefore setting the “heart rate” of the embryologic heart tube. Some of those cells eventually become the definitive SA node. The human heart is the first organ to develop; the earliest known contraction of the human heart at this stage occurs at around 23 days gestation (13). The heart begins formation in a region of the embryo known as the cardiac crescent. As the cylindrical heart tube forms from the cardiac crescent, the first peristaltic cardiac contractions occur (14). The first contractions are primarily calcium dependent and are mediated by NCX1 and the L-type calcium channel. These channels gradually form waves of calcium ions that are more and more synchronized (14). The sodium and potassium channels develop later in gestation (15). The pattern of gap junctions and ion channels, forming the mature “membrane clock” (16), then establishes the conduction speeds that will be characteristic of the future sinoatrial and atrioventricular nodes (12). The balance of transcriptional factors such as Nkx2-5, and transcriptional repressors like Tbx2, appears to cause the initial decision of whether or not a precursor cell becomes a definitive cardiomyocyte or a future conduction cell (17). Nkx2-5 is a homeobox gene, which is a category of genes responsible for regulating large-scale anatomic features. There are many downstream genes regulated by Nkx2-5, but two notable ones include Hand1 and Hand2, which are in large part responsible for the left-right asymmetry of the definitive heart (18). The separation between atrial cardiomyocytes and atrial conduction system is enforced by Nkx2-5, via its suppression of atrial cardiomyocytes and coordination with the Notch pathway (19). Tbx2 on the other hand, exerts one of its effects by suppressing atrial natriuretic peptide generation in the embryonic heart. This in turn controls the boundary between the atria and the ventricles in the future definitive heart (20). In a separate study, studies of embryos deficient in Tbx2 were found to have unbalanced atrioventricular canals (21).

## Common congenitally malformed hearts

### Common congenitally malformed hearts

The normal physiology of a few selected congenitally malformed hearts as well as their normal electrophysiology will be discussed next. Their surgical corrections are discussed in the last section.

The congenitally malformed heart results from errors in development at any of the normal stages of heart development (22). For example, the atrial septum is formed by complete septation of the two atria by two rims of tissue that are normally expected to span the gap between the atrial roof and the atrial floor. If these rims of septal tissue do not fully reach the tricuspid valve/mitral valve annuli for example, a primum atrial septal defect is formed. If the center of the heart, or crux, is not completely formed, any of the subtypes of the atrioventricular septal defect will occur. Normally, the primitive cardiac tube is supposed to fold a certain way to place the atria on top of the ventricles, and make the right ventricle connect to the right atrium. If this folding occurs in the reverse fashion, L-transposition of the great arteries can occur. This is a condition where the right atrium connects to the left ventricle and the right ventricle connects to the left atrium. Normally, the embryologic heart forms a single truncus arteriosus that is supposed to form the eventual definitive pulmonary artery/aorta, however a rotation and septation is supposed to occur for these two vessels to separate from each other. When this does not occur correctly, D-transposition of the great arteries can occur, where the right ventricle connects to the aorta and the left ventricle connects to the pulmonary artery. This condition is lethal in the absence of a location that can mix blood, as this circulation entirely separates the pulmonary and systemic circulations. Many different mixtures and permutations of these prior problems can then occur, creating more complex congenital heart lesions.

The most straightforward congenitally malformed hearts that pediatric cardiologists encounter are dysplasia of a single valve or an isolated septal defect. In the case of an isolated atrial or ventricular septal defect, surgical correction is straightforward, but the patient does sustain a nonzero risk of future arrhythmias (23). Specifically, the risk of atrial arrhythmia after atrial septal defect repair is worse the older the patient is, at date of surgical repair. In one Dutch study, patients with an atrial septal defect repair were assessed for 50 years after their original surgery. At the 50 year mark, the total survival was not much different from the general Dutch population, but the cumulative burden of symptomatic arrhythmias at this time point was 35% (24). In the patient post ventricular septal defect repair, some studies report varying degrees of cardiac limitation during exercise and right ventricular dysfunction (25). At least one long term outcome study reported the proportion of ventricular septal defect repair patients with symptomatic arrhythmias at 40 year followup as up to 13% (26). Therefore, most adult congenital clinics advise periodic followup to search for any significant arrhythmias that may result from scar lines associated with the prior surgery.

One of the most well studied congenital lesions is Tetralogy of Fallot. The corrective surgery involves correcting the ventricular septal defect, and the potentially multi-level pulmonic stenosis. The pulmonary valve, pulmonary arteries, and right ventricular outflow tract can all be affected in Tetralogy of Fallot, and so potentially, all three regions of the heart may require surgical correction to relieve stenosis. The long-term prognosis is most significantly altered when the pulmonary valve cannot be preserved during initial surgery, creating varying degrees of pulmonary insufficiency. This potentially requires correction with a later pulmonary valve replacement, which further commits the patient to more surgeries requiring more pulmonary valve replacements. Tetralogy of Fallot also has a unique ventricular arrhythmia burden even in those patients who have achieved complete correction, which requires ongoing monitoring. The most common ventricular arrhythmia mechanism in TOF is a macro-reentrant rhythm (27). Part of the ventricular arrhythmia burden is related to the ventricular septal defect repair, part of it is related to the chronic pressure/volume load of pulmonary insufficiency, and there is even emerging evidence that the right ventricle remains malformed at the cellular level despite complete surgical correction (28). Diastolic dysfunction of the left ventricle also negatively impacts right ventricular arrhythmia risk in adulthood (29).

Ebstein anomaly is a special type of congenitally malformed tricuspid valve, in which part of the tricuspid valve is adhered to the ventricular septum. This lesion carries varying degrees of severity. At a cellular level, the conduction system is also often malformed carrying a single or multiple additional diagonal accessory tracts (30) across the malformed tricuspid valve, setting up the patient for ventricular pre-excitation (Wolff-Parkinson-White syndrome) (31). Patients can receive surgical or catheter-based ablation of the accessory bypass tracts.

### Surgical techniques

Surgical corrections necessarily span the gamut of congenital heart lesions. The overall theme is to use patching, conduit placement, and certain more advanced techniques to reshape the malformed heart to something as close as possible to the normal human heart. Many techniques also require cardiac bypass, meaning the blood is temporarily removed from the heart but still circulated through the body by a machine. Foreign material inserted into the heart potentially forms boundaries of a future macro-reentrant circuit (32).

More complex surgeries such as correction of D-transposition of the great arteries, require coronary artery translocation, and translocation of the aorta and pulmonary artery (33). This carries both hemodynamic and potentially electrophysiologic long term consequences, especially if there is an additional ventricular septal defect requiring repair. The coronary suture lines place the patient at future risk of coronary stenoses, which present with a variety of cardiac symptoms. The suture line between the left ventricle and the aorta places the patient at risk for future aortic root dilation; while the precise mechanism is not completely worked out the leading hypothesis is that the pulmonary and aortic valves are different on the cellular level, with the native pulmonary valve staying in the systemic circulation. Given that the pulmonary valve is not designed to withstand systemic pressures, it can degrade over time and generate aortic root dilation. Patients with this diagnosis are at risk of multiple tachyarrhythmias and require ongoing monitoring for assessment.

## Epidemiology of sudden cardiac arrest

When assessing people with congenitally malformed hearts, up to 25% die due to sudden cardiac arrest, and when assessed across the entire population, it is 0.07–0.4 per 100,000 person years (34). When surveying the literature on deaths per year, when assessed across age groups and different palliated and non-repaired congenitally malformed hearts, the rate is 0.28%–2.7%, which is considerably higher than in the general population (34).

## Mechanisms of sudden cardiac arrest/arrhythmia in the congenitally malformed heart

### Mechanisms of right ventricular dysfunction in the congenitally malformed heart

With the probable exception of primary pulmonary hypertension, congenitally malformed hearts carry a much greater burden of right ventricular pressure/volume loading and subsequent dysfunction than adults with acquired heart disease. Due to the unique balance of Ito/Iks channels, the refractory period of the RV is shorter, making it more vulnerable to arrhythmias (35). Depending on the specific congenital heart lesion, this risk is further modified by hypoxia, genetic factors, and conducted atrial arrhythmias (36). Differences in potassium, calcium, and sodium ion handling in the remodeling right ventricle can predispose the congenitally malformed heart to more ventricular arrhythmias (37–39). While the evidence for this in adults is only emerging, laboratory evidence for altered remodeling and decreased connexin density may also contribute (40). When conduction heterogeneity exists, this can be identified by the presence of a fragmented QRS pattern, which is reported to correlate with the risk of future sudden cardiac arrest (35).

### Electrophysiological dysfunction in atrial switch and heterotaxy

The atrial switch and single ventricle surgeries present a unique set of arrhythmia challenges to the cardiologist and electrophysiologist (23). The atrial switch is an older surgery meant to correct D-transposition of the great arteries before the more modern arterial switch could be done safely. Such patients are reaching their 40–50s as of 2024, and due to the presence of a systemic right ventricle, they all have varying degrees of HFpEF and HFrEF. The patch used to form the baffle that switches the atria has very extensive suture lines (23), which set up the baffle for leaks and multiple atrial arrhythmias. The pressure and volume overloaded right ventricle slowly generates fibrosis over time, and is susceptible to malignant ventricular arrhythmias. Typically this surgery is only done in the modern operating suite as part of a complicated double switch procedure for the anatomical correction of levotransposition of the great arteries in selected cases (41).

Heterotaxy describes the presence of a situs other than the normal trilobed right lung, levocardia, dual lobed left lung, and left sided stomach. The heart need not carry the same situs as the other viscera, indeed one of the simplest kinds of heterotaxy is mirror image situs inversus, meaning the organs are on the typical side of the body, but the heart is inverted. In the most complex kinds of heterotaxy, arrhythmias can be triggered by the presence of dual SA nodes, dual AV nodes, or the absence of an SA node altogether (42).

### Electrophysiological dysfunction in L-transposition

L-transposition of the great arteries has a wide spectrum of associated lesions, however the peculiar conduction system is uniquely predisposed to heart block, with the absolute risk rising with each decade of adulthood. Other than monitoring for ventricular dysfunction of the systemic right ventricle (in cases of straightforward L-TGA), patients require monitoring to determine when they might be in need of a pacemaker to correct symptomatic bradyarrhythmias, which can be further complicated by unusual coronary sinus anatomy should a coronary sinus lead be required (43).

### Electrophysiological dysfunction in the single ventricle palliation

**Figure 2:**
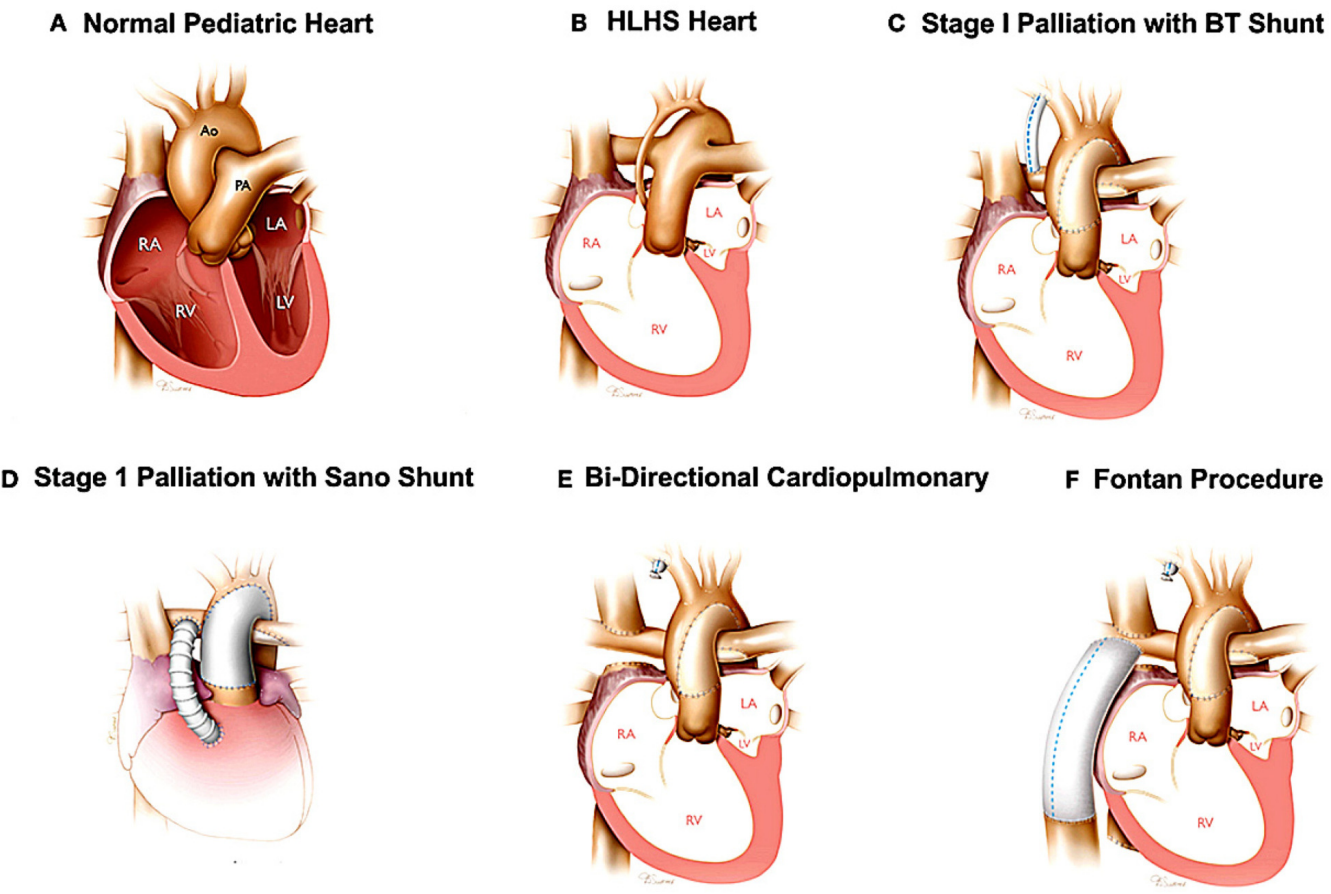
The Fontan procedure (in this case illustrated as a palliation of hypoplastic left heart syndrome). The additional surgeries required before the Fontan are also depicted. Panel **A** illustrates a structurally normal heart. Panel **B** illustrates the hypoplastic left heart. Panel **C** and **D** illustrate the two potential surgeries that can be used as the first stage palliation of HLHS. Panel **E** illustrate the second stage palliation. Panel **F** illustrates the Fontan surgery, or total cavopulmonary anastomosis. Reproduced with permission from “Surgical reconstruction of HLHS hearts” by Birla et al., licensed under CC BY 4.0.

The single ventricle surgeries, culminating in a total cavopulmonary anastomosis, present unique hemodynamic and electrophysiological challenges (Figure 2). The surgery was invented in the 1980s to help patients with less than two functioning ventricles survive, and requires that the superior vena cava, inferior vena cava, right pulmonary artery, and left pulmonary artery be connected to each other in stages. Because the atria will have multiple scar lines and fibrosis by the time all staged surgeries are complete, it is susceptible to multiple macro-reentrant rhythms. Given the presence of surgery close to the SA node, the Fontan is uniquely susceptible to sinus node dysfunction far earlier than adults with acquired heart disease. The Fontan circulation itself is also susceptible to multiple hemodynamic issues that are described elsewhere (44).

### Effect of exercise on arrhythmia risk in the adult congenital patient

Exercise has a variable effect on arrhythmia risk in the congenitally malformed heart. While there is general agreement that moderate exercise is cardioprotective in most cases, certain kinds of exercise carry higher risk.

Certain congenital heart diseases carry a unique burden of arrhythmia during exercise. Hypertrophic cardiomyopathy, significant aortic valve stenosis, congenital coronary anomalies, and cyanotic congenital heart disease are the major categories.

In aortic valve stenosis and congenital coronary anomalies, some mechanisms of sudden cardiac arrest are shared. The stenotic aortic valve can generate left ventricular hypertrophy, diastolic dysfunction, and eventual arrhythmias related to obstruction (45). In the anomalous coronary, abnormal takeoffs can result in less than normal size coronary orifices, which can generate ischemia and subsequent arrhythmia during periods of exercise as the orifice is compressed (46).

In hypertrophic cardiomyopathy, one of the mechanisms for hypertrophy is cardiomyocyte disarray with abnormal sarcomere organization. Sudden cardiac arrest can result from the cardiomyocyte disarray, which may set up re-entrant pathways. Alternatively, significant left ventricular outflow tract obstruction may occur (47).

In cyanotic congenital heart diseases, two major mechanisms of cardiac arrest include paradoxical emboli and systemic steal (48). In rare circumstances, right ventricular cardiomyopathy can evolve rapidly after atrial septal defect closure (49).

In Tetralogy of Fallot, a sustained exercise program of moderate aerobic exercise was found to decrease the risk of ventricular arrhythmias (50). In a group of 170 patients with corrected congenital heart lesions using a right ventricular-pulmonary artery conduit, the incidence of provoked ventricular arrhythmias during all phases of exercise was 44% (51). However, in the same study, these ventricular arrhythmias were not correlated with significant hemodynamic impairment. One of the most well established high risk categories is arrhythmogenic cardiomyopathy; in this group of patients high intensity exercise is not recommended due to risk of life threatening arrhythmias (52). In a 2024 nationwide Danish cohort study done over 47 years, the complexity of the repaired heart lesion was correlated with the frequency of the arrhythmia, with Tetralogy of Fallot, transposition of the great arteries, and the atrioventricular septal defect ranking amongst the highest risk. The strongest evidence supporting arrhythmia risk during exercise comes from those patients with residual documented ventricular arrhythmias or residual significant hemodynamic lesions (53), but studies are more sparse on the precise arrhythmia risk stratified by repaired congenital heart disease.

### Electrophysiological dysfunction in channelopathies, arrhythmic MVP, and common genetic disorders with arrhythmic consequences

Multiple channelopathies have pediatric counterparts, such as long QT syndrome, short QT syndrome, Brugada syndrome, and catecholaminergic polymorphic ventricular tachycardia (even in the otherwise structurally normal pediatric heart). The mechanisms of arrhythmia generation are the same, but fewer prospective and randomized clinical trials are available to guide detailed risk stratification in children. Some class I criteria are extrapolated from adult EP guidelines, such as the presence of resuscitated sudden cardiac arrest. Device selection in adults can typically lean towards the transvenous ICD, however the potential for considerable somatic growth in children necessitates the consideration of epicardial or subcutaneous devices. Furthermore, children carry higher rates of device complications (54), making detailed pre-implantation counseling especially important.

The arrhythmic mitral valve prolapse disease is an association of mitral valve prolapse with ventricular arrhythmias. Several known mechanisms contribute to arrhythmogenesis in this condition. Myxomatous mitral valve degeneration may occur (55), which may generate abnormal stretch of the mitral valve ring. This repetitive mechanical stress can generate scarring of the left ventricular mass, which can generate a focus for ventricular tachycardia. Scarring can be identified on cardiac MRI, and can guide the decision on when such a patient may require an ICD. The inheritance pattern is primarily autosomal dominant with variable expressivity, but this appears to not be a single gene disease (56). The preferred method to screen children of parents with arrhythmic MVP remains to be seen.

Finally, multiple genetic mechanisms exist that modify the risk of arrhythmia in the congenitally malformed heart. While not all congenital heart lesions have known genetic causes, several syndromes exist with distinct genetic risk factors, such as Holt Oram syndrome, Kearns-Sayre syndrome, arrhythmogenic cardiomyopathy, and DiGeorge syndrome. NKX2.5 is a transcription factor necessary for the activation and maintenance of the cardiac regulatory network during embryologic development (57). Depending on the mutation, abnormal NKX2.5 may be associated with atrioventricular block, atrial fibrillation, SVT, or ventricular arrhythmias. Holt-Oram syndrome is caused mostly by mutations in the TBX5 gene, which is a transcription factor controlling the formation of the atrial septum and conduction system. Mutations in TBX5 are capable of causing atrioventricular block. Digeorge syndrome is caused by a 22q11 deletion, which is capable of causing hypocalcemia along with congenital heart defects. Hypocalcemia may cause a variety of arrhythmias, and for this reason, is typically aggressively replaced. Patients with Tetralogy of Fallot and Digeorge syndrome, a common combination, are at risk of arrhythmias from both conditions (58). Genetic testing is thus very useful in aiding the clinician in further risk stratifying the patient with the congenitally malformed heart from the arrhythmia perspective. This type of testing can easily be obtained by buccal smear and processing at a reference genetics lab (59). Clinical, histological, and molecular autopsy should be used together to provide a diagnosis that is as accurate as possible for the sudden cardiac arrest patient as well as family members (60).

## Current treatments

The most direct treatments of sudden cardiac arrest are advanced cardiac life support in the moment, defibrillation for a shockable rhythm, and prior placement of pacing for symptomatic bradycardic rhythms. Additional therapies extrapolated from acquired heart disease are also being attempted in adult congenital heart disease (61). The discussion that follows is a selection of electrophysiological issues that are unique to congenital heart disease from a few representative lesions with special considerations.

The sequential steps taken to treat and prevent arrhythmias with the congenitally malformed heart generally follow those of patients with acquired heart disease, with some notable exceptions that are specific to the congenital heart lesion being considered. The overarching principle is that a structural problem, when not adequately addressed (such as the diagnosis of a coarctation or pulmonary valve insufficiency), may create an adverse hemodynamic load and eventual arrhythmias. A monitoring device may be the first step to assessing a patient with dizziness or palpitations, to determine what sort of arrhythmia may be present. The specific medication that is given for antiarrhythmic control typically varies slightly depending on the clinician, but typically follows a gradient from choosing an agent with the least side effects first and gradually increasing dosage and number of medications depending on how difficult the arrhythmia is to control. Electrophysiological studies may be considered for macro-reentrant rhythms for more definitive control and the possibility of being able to use less antiarrhythmic medication. Pacing is typically undertaken for traditional criteria like symptomatic bradycardia, and implantable cardioverter-defibrillators are implanted for primary and secondary prevention typically following standardized criteria (53). Some notable exceptions are described next.

Kearns-Sayre syndrome is the triad of cardiac conduction disturbances, progressive external ophthalmoplegia, and pigmentary retinopathy. The syndrome is caused by a mitochondrial mutation in the genes responsible for oxidative phosphorylation (62). Because of the rapid progression to hemodynamically unstable complete heart block, such patients are referred for early pacemaker placement. Survival to adulthood in this syndrome carries significant challenges and sometimes requires cardiac transplant (63).

**Figure 3:**
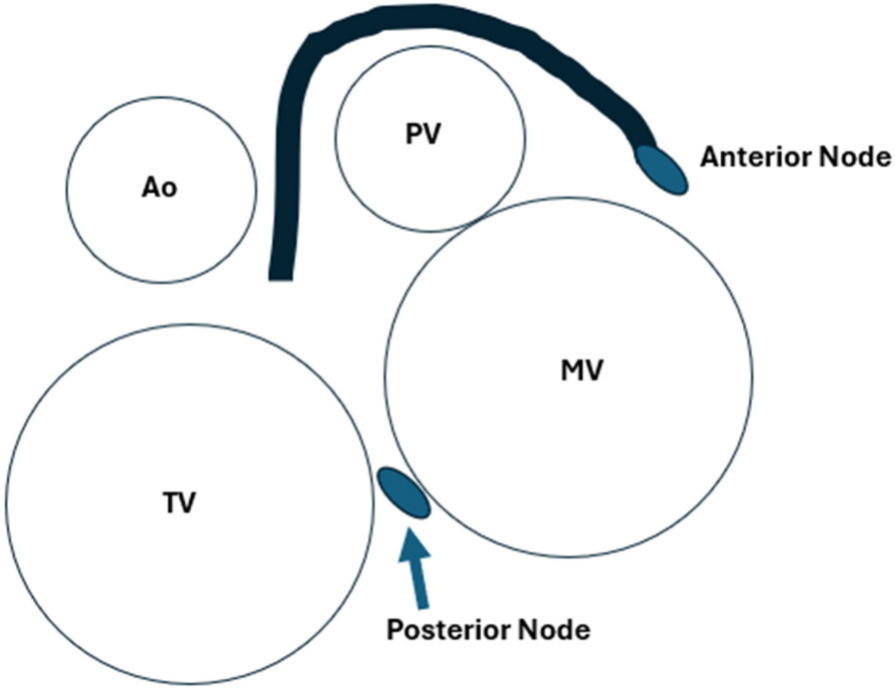
The approximate position of the dual AV nodes and conduction system of L-TGA from the point of view of the atrioventricular valves, with normal atrial situs.

Levo-transposition of the great arteries (L-TGA) has been described above, but its most significant conduction system complication is the increasing risk of heart block with each decade of life. Typically, there are two major configurations of the conduction system in L-TGA. When usual atrial arrangement L-TGA occurs, there is an anterior AV node anterior and to the right of the mitral valve, with the His bundle crossing in front of the pulmonary valve and then forming the right and left bundles. There is also a secondary AV node that is posterior to the His bundle. When mirror image L-TGA is present, the conduction system is mostly contained in the anatomic ventricular septum similar to the normal heart, although a secondary AV node is still present anteriorly (64) (Figure 3). When the ventricular septum carries a large septal defect, the right and left bundles are further distorted and avoid the VSD. At the cellular level, the abnormally increased length of the conduction bundles and the propensity for early fibrosis explains the progressive increase in risk of heart block.

The pathophysiology of Tetralogy of Fallot has been described in previous sections but a unique criteria predisposing patients to sudden cardiac arrest is a QRS duration >180 ms (65). This criteria is used with other traditional clinical criterion, such as cardiac arrest or ventricular tachycardia, to decide when such a patient should receive an implantable cardioverter-defibrillator.

The absolute most important actions to take are preventative. The major actions that can be taken differ by age group (66). A four-fold reduction in sudden cardiac arrest mortality in newborn infants can be achieved if universal fetal echocardiogram screening is implemented (67, 68). In older children, targeted screening programs for common conditions associated with sudden cardiac arrest can help early detection (69–72). Sudden cardiac arrest takes special significance in the pregnant mother, as risk stratification will be relevant to both the mother and fetus. Some conditions such as Marfan syndrome, carry higher risk once pregnancy occurs (73).

## Future directions

### Advances in medical management

Much of the medical management geared towards heart failure in congenital heart disease mirrors the management of heart failure in acquired heart disease or is extrapolated. Afterload reduction, diuresis, and MRA therapy remain the mainstays of ACHD (grown up congenital/adult congenital heart disease) heart failure management although specific evidence for specific lesions may remain sparse. SGLT2 inhibitors have shown promise in preventing heart failure hospitalization in the ACHD population (74). While the ability to prevent sudden cardiac death in adult congenital heart disease has greatly improved in the past decades, it remains harder to prevent when the root cause is due to pulmonary hypertension (75).

### Pulmonary hypertension

A few notable lesions do generate a PH risk on top of the risks already inherent to the congenital heart lesion at hand. Certain significant left to right shunts such as atrial septal defects, can present in heart failure with pulmonary hypertension if they are diagnosed late (23). Tetralogy of Fallot with multiple aortopulmonary collateral arteries may also generate PH even many decades after initial corrective surgery. Traditional PH therapies are used such as PDE-5 agents, prostacyclin analogs, and ERA agents, with similar dosing and monitoring regimens as in the structurally normal heart (76). Newer agents and repurposed drugs such as imatinib and sotatercept, are being tested to try and advance the field (77, 78).

### Predictive scores

Attempts at more comprehensive predictive scores have been done with some success, such as the Prevention-ACHD study (79). The score assigns 1 point each to coronary artery disease, New York Heart Association class II/III heart failure, supraventricular tachycardia, systemic ejection fraction <40%, subpulmonary ejection fraction <40%, QRS duration ≥120 ms, and QT dispersion ≥70 ms. When the total score is 3 or more, high arrhythmia risk is predicted, and this was found to be superior to standard HRS indications for placing an ICD/pacemaker.

A score intended to help predict risk of cardiac arrest in children with HCM is the PRIMACY score. The components of this score include the age, LV wall thickness z-score, LA z-score, LVOT gradient, history of unexplained syncope, presence of NSVT, and the genotype of the patient. For HCM, the positive predictive value of the PRIMACY score has been found to be lower in a French study, and so it is recommended to use such scores in conjunction with other factors (80).

### Advances in cardiac imaging

Cardiac MR can give multiple pieces of additional information that place it as the strongest noninvasive tool for tissue characterization. The major additional pieces of added information include tissue characterization, scar quantification, and edema quantification. The ability of cardiac MRI has been assessed both in congenital and acquired heart disease. For example, adults with single ventricles and a Fontan palliation have multiple pathways that may lead to failing Fontan physiology and at times, sudden cardiac arrest. Cardiac MRI has improved in its ability to predict sudden cardiac arrest in this population (81). In over 3,000 patients, which spanned both children and adults with a Fontan, ventricular dilation, PLE, EF < 50, and NYHA class >II were predictive of ventricular arrhythmia, with people lacking those risk factors having >99.5% event free survival.

In pediatric hypertrophic cardiomyopathy, 700 patients were assessed across a 7 year period via cardiac MRI to define the percent late gadolinium enhancement and its association with sudden cardiac arrest. People with a burden of LGE > 10% were more likely to have sudden cardiac arrest (82).

In Tetralogy of Fallot, the primary use of cardiac MRI is to guide the replacement of the pulmonary valve in addition to the typical hemodynamic data it provides. Given the high incidence of RV systolic and diastolic dysfunction, MRI is also important in determining the presence of microscopic fibrosis and overt scarring. Scar quantification aids the clinician in risk stratification for sudden cardiac arrest. In a recent study of 3 dimensional late gadolinium enhancement, cardiac MRI was able to identify slowly conducting anatomic isthmuses to improve noninvasive risk stratification (83). Other centers are attempting to define the role of MRI assessment of diastolic dysfunction in Tetralogy of Fallot for the future prediction of adverse cardiac outcomes (84).

### Advances in device therapy

Given the much longer time span that children and young adults are expected to need implanted devices, more discussion of risk/benefit is required, as the potential period of exposure to adverse events is greater than in adults. According to a retrospective nationwide cohort study in Denmark (85), during a 39 year follow-up period, 72 children were found to have received an ICD, and most had channelopathies or a congenitally malformed heart. Most were done for secondary prevention, and most children experienced more therapies and more complications than a comparable cohort of adults.

While it has not been widely used in the world of adult congenital heart disease, the internally implanted cardiac monitor AngelMed Guardian ® has been tested in a case study of a patient with ischemia related to coronary myocardial bridging (86). It is plausible that additional congenital anomalies could take advantage of this tool. The implantable loop recorder is currently available from several vendors for this purpose.

Leadless pacemaker devices now have quite a bit of background research in acquired heart disease, but remain new to the adult congenital world. In patients who are high risk for epicardial pacemaker placement, the leadless pacemaker is a plausible option when those patients are acceptable risk for transcatheter intervention (87).

When an ICD is required, patients carry several options including the traditional transvenous ICD, the EVICD, and the subcutaneous ICD. Anatomical considerations such as unusual baffle anatomy, may push the operator to select the extravascular ICD over the traditional ICD especially if there is a stenosed or hypoplastic superior vena cava. When CRT-D is required, certain anatomy may not be amenable to traditional left ventricular pacing through the coronary sinus, especially when the CS is hypoplastic or absent such as is common in levotransposition of the great arteries. While both the EVICD and subcutaneous ICD do not require entry into the vascular system, the EVICD has the major advantage of being able to conduct antitachycardia pacing as well as defibrillation. More recent advancement in biomedical engineering is attempting to eliminate leads entirely by creating the leadless ICD, which remains in the clinical trial stage (88).

### Advances in gene therapy and surgical therapy

CRISPR-Cas13 therapy has successfully prevented cardiac hypertrophy in a humanized mouse model of a specific subtype of HCM (89). The rationale behind this CRISPR technique, is that MYH7 cardiomyopathy exerts a dominant negative effect on cardiac contractility, and CRISPR is meant to suppress the defective allele. In normal cardiomyocytes, MYH7 forms the beta myosin heavy chain. This group delivered an optimized CRISPR system via an AAV9 vector to a mouse model of human MYH7 HCM, and found statistically significant improvements in ventricular thickness.

The clinician treating adult congenital heart disease patients should maintain a high degree of suspicion for hemodynamic lesions when arrhythmias occur. For example, a dilated right ventricle is not expected to spontaneously resolve in the face of severe pulmonary insufficiency (90). Should that same patient carry recalcitrant atrial flutter, the atrial arrhythmia burden is not expected to improve until the pulmonary insufficiency is corrected. Sympathetic denervation has been studied in the acquired heart disease population and long QT populations, but its utility in ACHD is less well studied.

In conclusion, congenital heart lesions have a great deal of physiological and electrophysiological issues which may lead to sudden cardiac arrest. In the past few decades, many advancements have been made in prevention of sudden cardiac arrest in the adult congenital population, but knowledge gaps exist as well as a gap in the amount of preventable sudden cardiac arrest compared to the acquired heart disease population. Medications, pacemakers, and defibrillators remain the mainstay of cardiac arrest prevention, as well as typical lifestyle counseling. However, the wide spectrum of congenital heart lesions introduces unusual idiosyncrasies that affect their arrhythmia management such as unique propensities for tachyarrhythmias and bradyarrhythmias. Future treatments involve better risk stratification, repurposing of prior medications, and the testing of novel arrhythmia monitoring devices.
